# Up-Converting Nanocrystals Modified With Fluorescent Markers for the Detection of Amino Acids: Preparation, Characterization, and Sensing Performance

**DOI:** 10.3389/fchem.2022.859963

**Published:** 2022-03-21

**Authors:** YuLang Fei, Kun Wu, Liang Liu

**Affiliations:** ^1^ Medical College, Xijing University, Xi’an, China; ^2^ School of Materials Science and Engineering, Jiangsu University, Zhenjiang, China; ^3^ Key Laboratory of Advanced Functional Materials and Devices of Anhui Province, Hefei University of Technology, Hefei, China

**Keywords:** up-converting nanocrystals, rhodamine molecules, cysteine, luminescence sensing, emission

## Abstract

The present work was devoted to developing rhodamine-like chemosensing systems for cysteine (Cys) optical recognition. Aiming at low background light and minimal photobleaching effect, up-converting nanocrystals were firstly synthesized and latterly coated by α-cyclodextrin, and finally used as an exciting host. An energy transfer procedure from these nanocrystals and rhodamine sensors was established *via* their spectroscopic analysis and emissive decay dynamics comparison. The binding dynamics of our chemosensors for Cys were revealed to have uncomplicated recognition with a stoichiometric ratio of 1 *vs.* 1. The addition of cysteine increased the emission intensity of the chemosensors. As a consequence, the luminescence off-on effect with sensing selectivity and linear sensing behavior for Cys was demonstrated. Sulfur modification on our chemosensors was shown to be effective in improving their selectivity and photostability.

## 1 Introduction

Their participation in biological activities makes amino acids critical human health factors. Cysteine (Cys) is an attractive amino acid since its abnormal level has been connected to human diseases, including skin damage, growth hypoevolutism, bowel disease, etc. (Seshadri et al., 2002; Wald et al., 2002; Levine et al., 2008; Wang et al., 2014a). Optical sensing has been demonstrated to be an effective analytical technique owing to its quick signaling, non-invasive detection, and limited instrumental demand (Wang et al., 2012; Wang et al., 2014a; Wang et al., 2014b). To achieve suitable performance and specific features, a composite structure is highly expected in these chemosensing systems since this composite structure unifies features from every individual component and easily satisfies demands for real-world applications (Wang et al., 2014b; Lim et al., 2014). There are two functional components in a hybrid structure, which are the chemosensor and its supporting material. The former component ensures target signaling, while its supporting substrate disperses and holds this chemosensor so that minimal chemosensor self-quenching and fluent target diffusion can be realized (Zhang et al., 2007; Awual et al., 2015; Chu and Chuang, 2015). For example, Chen and Sun have reported a promising photodynamic therapy using a water-soluble aggregation-induced emission photosensitizer activated by an acidic tumor microenvironment (Min et al., 2002). Additionally, there are ligand-triggered Pt(II) metallacycles with mechanochromic and vapochromic responses which could find wide and attractive application in biological and mechanical sensing fields (Chen et al., 2019; Yin et al., 2021). More optoelectronic systems have been reported for chemosensing applications (Chen et al., 2021a; Chen et al., 2021b; Sun et al., 2021).

As for chemosensors, they usually suffer from limited photostability since they require high-energy excitation light such as UV radiation (Lim et al., 2014; Awual et al., 2015; Chu and Chuang, 2015). An alternative method is using up-converting materials as an exciting source. In other words, the up-converting exciting source absorbs IR photons and then converts their energy to the signaling component, without decomposing the chemosensor structure (Liu et al., 2008; Zhang et al., 2012). As a charming option, an NaYF_4_ lattice is constantly applied based on its advantages of good quantum yield, prime uniformity, limited biotoxicity, and considerable spectroscopic matching for biological windows (Li and Zhang, 2006; Liu et al., 2008; Zhou et al., 2012; Peng et al., 2014). Oleic acid was selected to stabilize the NaYF_4_ lattice during NaYF_4_ preparation which coated the resultant NaYF_4_ lattice and made it hydrophobic (Peng et al., 2014). To improve their water-dispersibility, the NaYF_4_ lattice can be modified by a phase transfer with α-cyclodextrin (α-CD). Its self-assembly reaction allows for a microenvironment with a hydrophilic edge and a hydrophobic cage. The hydrophobic oleic acid is bonded with the α-CD cage. While the α-CD hydrophilic edge is open to the surrounding environment (Dujols et al., 1997; Peng et al., 2014). As a consequence, the resultant NaYF_4_ lattice can be made hydrophilic.

**SCHEME 1:**
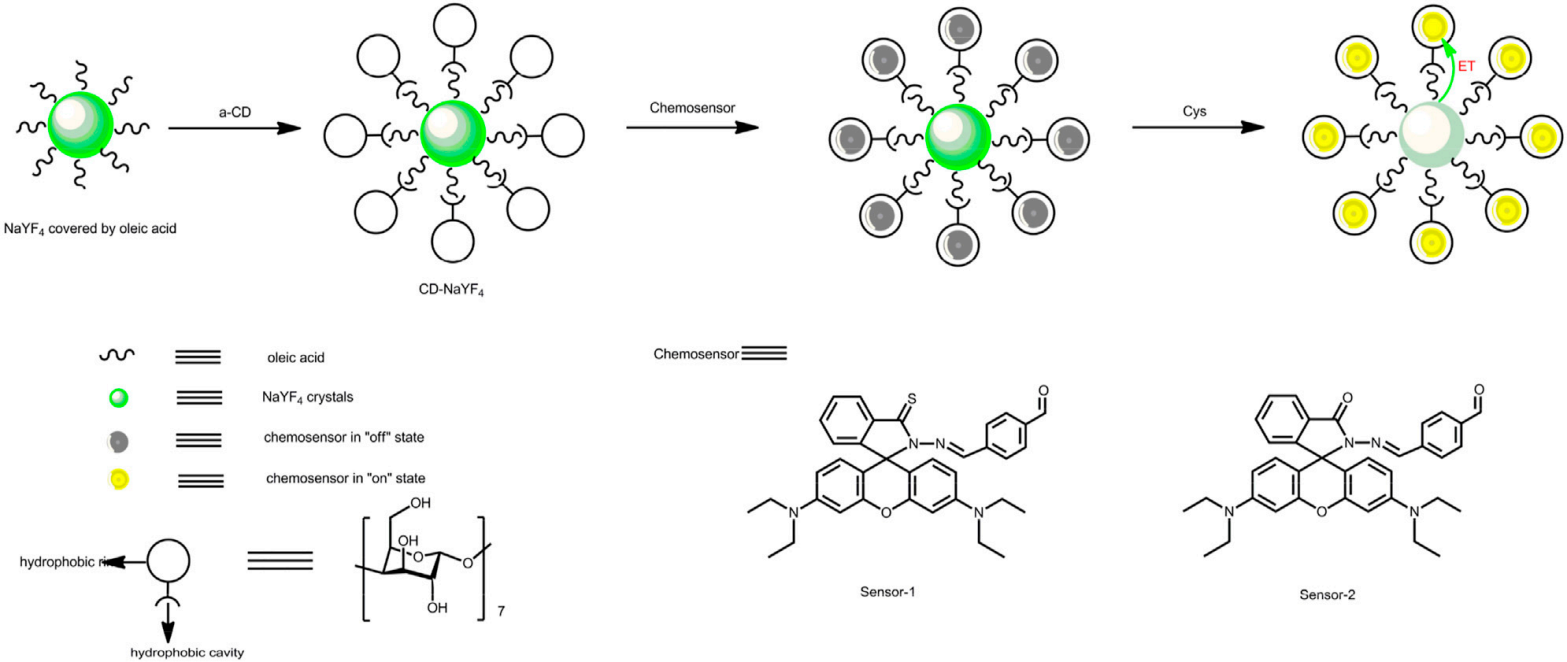
Design strategy of CD-NaYF_4_, Sensor-1, and Sensor-2.

Enlightened by the above discussion, in the following work, rhodamine-derived chemosensors are designed and synthesized, as demonstrated by Scheme 1. Up-converting NaYF_4_ nanocrystals are prepared, modified by cyclodextrin, and then applied as the exciting source to improve the photostability of our chemosensors. The composite structure of the up-converting excitation lattice and rhodamine-derived chemosensors is anticipated to have high sensitivity, good selectivity, and improved photostability.

## 2 Experimental Section

### 2.1 General Information

Untreated starting chemicals, such as rhodamine reagent, terephthalaldehyde, Lawesson’s reagent (LR), and cyclodextrin, were supplied by Donghu Chemical Company (Tianjin) and used for synthesis. Some common products, including POCl_3_, C_2_H_5_OH, anhydrous NH_2_NH_2_ (90 wt%), 1-octadecene, oleic acid (OA), cyclohexane, acetonitrile, *n*-hexane, and RE salts, were purchased from Souxian Chemical Company (Shanghai). Organic solvents and solvent water were redistilled.

Composite sample powder was diluted by phosphate buffer (PBS, pH = 7.0) and treated by ultrasonification for 300 s. The concentration was 5 mg in 10 ml. Micromorphology was provided by a Hitachi S-4800 microscope and a JEM-2010 transmission electron microscope. NMR data were collected using a Varian INOVA 300 spectrometer. MS data were collected by an Agilent 1100 MS spectrometer. IR spectra were collected by a Bruker Vertex 70 FTIR spectrometer (KBr). Photophysical spectroscopy and dynamics were performed on a Shimadzu UV-3101PC spectrophotometer, a Hitachi F-7000 spectrophotometer, and a TEKTRONIX TDS-3052 oscilloscope. A 980 tunable laser was applied as the exciting light.

### 2.2 Synthesis of Sensor-1 and Sensor-2

The Sensor-1 synthetic strategy is explained as follows (Dujols et al., 1997). The following reagents were added into a flask, including rhodamine B (20 mmol), CHCl_3_ (15 ml), and POCl_3_ (10 ml). Then the mixture was stirred at 25°C for 25 min and at 90°C for 12 h under an N_2_ atmosphere. Solvent and excess POCl_3_ were extracted by thermal evaporation under decreased pressure. CH_3_CN (110 ml) and anhydrous NH_2_NH_2_ (11 ml) were mixed into the above product and stirred at 25°C for 1 h and at 91°C for another 12 h under an N_2_ atmosphere. Solvent and excess NH_2_NH_2_ were extracted by thermal evaporation under reduced pressure to give rhodamine B hydrazide. ^1^H NMR (CDCl_3_), *Δ* (ppm): 1.16 (t, 12H, NCH_2_ *CH*_3_, *J* = 6.9 Hz), 3.23 (q, 8H, N*CH*_2_CH_3_, *J* = 6.9 Hz), 3.61 (s, 2H, N-*NH*_2_), 6.22 (dd, 2H, Ar-H. *J* = 2.4 Hz, *J* = 9.0 Hz), 6.36–6.39 (m, 4H, Ar-H), 7.18–7.19 (m, 1H, Ar-H), 7.46 (t, 2H, Ar-H, *J* = 3.9 Hz), 7.89 (m, 1H, Ar-H). EI-MS *m*/*e*: calc. for C_28_H_32_N_4_O_2_, 456.2; found, 456.8 [m]^+^.

Then the obtained product was reacted with LR by the following procedure (Wang et al., 2011). A mixture of rhodamine B hydrazide (10 mmol) and LR (12 mmol) in anhydrous toluene (30 ml) was prepared. It was heated at 120°C for 6 h under an N_2_ atmosphere. Then toluene was extracted by thermal evaporation. The solid product was chromatography-purified to give sulfur-substituted rhodamine B hydrazide. Silica gel column, eluent = petroleum: CH_2_Cl_2_ (30:1). ^1^HNMR (CDCl_3_), *δ* (ppm): 1.21–1.23 (t, 12H, NCH_2_ *CH*_3_), 3.29–3.32 (q, 8H, N*CH*_2_CH_3_), 3.79 (s, N-*NH*_2_), 6.14 (s, 2H, xanthene-H), 6.41–6.43 (m, 4H, xanthene-H), 7.22–7.24 (dd, 1H, Ar-H), 7.59 (dd, 2H, Ar-H), 8.05 (dd, 1H, Ar-H). MS *m*/*z*: calc. for C_28_H_32_N_4_OS, 472.2; found, 472.8 [m]^+^.

Finally, a mixture of the above prepared sulfur-substituted product (4 mmol) and terephthalaldehyde (5 mmol) in C_2_H_5_OH (60 ml) was heated at 80°C for 12 h (Cui and Zhang, 2014). C_2_H_5_OH was extracted by thermal evaporation. Solid residue was chromatography-purified to give Sensor-1. ^1^H NMR (CDCl_3_), *δ* (ppm): 1.19 (t, 12H, NCH_2_ *CH*_3_), 3.43 (q, 8H, N*CH*_2_CH_3_), 6.36 (dd, 2H, xanthene-H), 6.31 (d, 2H, xanthene-H), 6.49 (d, 2H, xanthene-H), 7.25 (m, 2H, Ar-H), 7.57 (dd, 2H, Ar-H), 7.69 (d, 2H, Ar-H), 7.88 (d, 2H, Ar-H), 8.11 (dd, 1H, Ar-H), 9.70 (d, 1H, −CHO). ^13^C NMR (CDCl_3_), *δ* (ppm): 12.34, 44.72, 58.49, 68.81, 97.49, 106.29, 108.71, 121.38, 124.47, 127.82, 128.42, 129.35, 133.64, 136.77, 142.73, 145.38, 148.83, 151.64, 153.29, 171.83, and 191.62. ESI-MS *m*/*e*: calc. for C_36_H_36_N_4_O_2_S, 588.3; found, 588.9 [m]^+^.

Sensor-2 was prepared similarly, but rhodamine B hydrazide was used for this route. ^1^H NMR (CDCl_3_), *δ* (ppm): 1.22 (t, 12H, NCH_2_ *CH*_3_), 3.38 (q, 8H, N*CH*_2_CH_3_), 6.33 (dd, 2H, xanthene-H), 6.41 (d, 2H, xanthene-H), 6.51 (d, 2H, xanthene-H), 7.19 (m, 2H, Ar-H), 7.57 (dd, 2H, Ar-H), 7.71 (d, 2H, Ar-H), 7.88 (d, 2H, Ar-H), 8.14 (dd, 1H, Ar-H), 9.62 (d, 1H, −CHO). ^13^C NMR (CDCl_3_), *δ* (ppm): 12.72, 44.38, 58.84, 66.51, 97.58, 106.84, 108.47, 123.62, 124.52, 127.26, 128.74, 129.86, 133.46, 136.63, 141.81, 145.53, 148.24, 151.73, 153.25, 164.47, and 191.22. ESI-MS *m*/*e*: calc. for C_36_H_36_N_4_O_3_, 572.3; found, 572.8 [m]^+^.

### 2.3 Synthesis of Up-Converting Lattice and its Cyclodextrin Coating

Our up-converting lattice was prepared according to a reported route and then modified with α-CD (Xie et al., 2009; Boyer and van Veggel, 2010; Wang et al., 2011; Cui and Zhang, 2014; Zou et al., 2014). ErCl_3_·6H_2_O (0.04 mmol) was mixed with YbCl_3_·6H_2_O (0.40 mmol). Then YCl_3_·6H_2_O (1.56 mmol) was added. 1-octadecene (30 ml) and oleic acid (12 ml) were mixed then finally added. This mixture was heated at 160^o^C for 35 min. Finally, NaOH (5 mmol) was slowly added, followed by the addition of CH_3_OH (20 ml) and NH_4_F (8 mmol). This mixture was heated at 75°C for 35 min, 100°C for 35 min, and 300°C for 60 min. At last, 100 ml of ethanol was mixed together. The powder sample was diluted with hexane. The doping ratio was 5 mg in 10 ml. Then the α-CD solution (5 mM) was added (1:1) and stirred for a whole day. The water phase was collected by centrifuge to yield the up-converting lattice coated in cyclodextrin (denoted as CD-NaYF_4_).

## 3 Results and Discussion

### 3.1 Design and Synthesis of Sensor-1, Sensor-2, and CD-NaYF_4_

For a clear understanding on our excitation host and chemosensors, their design strategy and synthesis are explained below. The conformational transformation between spirolactam geometry (no emission) and ring-open xanthene geometry (fluorescent) makes rhodamine-like molecules promising chemosensors with fluorescence turn-on characteristics (Xie et al., 2009; Wang et al., 2011; Cui and Zhang, 2014; Zou et al., 2014). In this work, rhodamine hydrazide was connected with terephthalaldehyde, in order to construct a sensing recognition site towards cysteine (Wang et al., 2011). The original rhodamine hydrazide was reacted with Lawesson’s reagent, aiming at improved sensitivity. For an optimal spectral overlap between chemosensor absorption (∼550 nm) and host emission, Yb(III), and Er(III) ions were selected, serving as energy-acceptor and emitter in NaYF_4_, respectively (Wang et al., 2011; Cui and Zhang, 2014; Zou et al., 2014). This NaYF_4_ lattice was then modified by cyclodextrin to increase its aqueous compatibility, due to the hydrophilic edge and hydrophobic cage in cyclodextrin. The composite structure of the up-converting excitation lattice and rhodamine-derived chemosensors is anticipated to have high sensitivity, good selectivity, and improved photostability.

### 3.2 Characterization Analysis of CD-NaYF_4_

**FIGURE 1:**
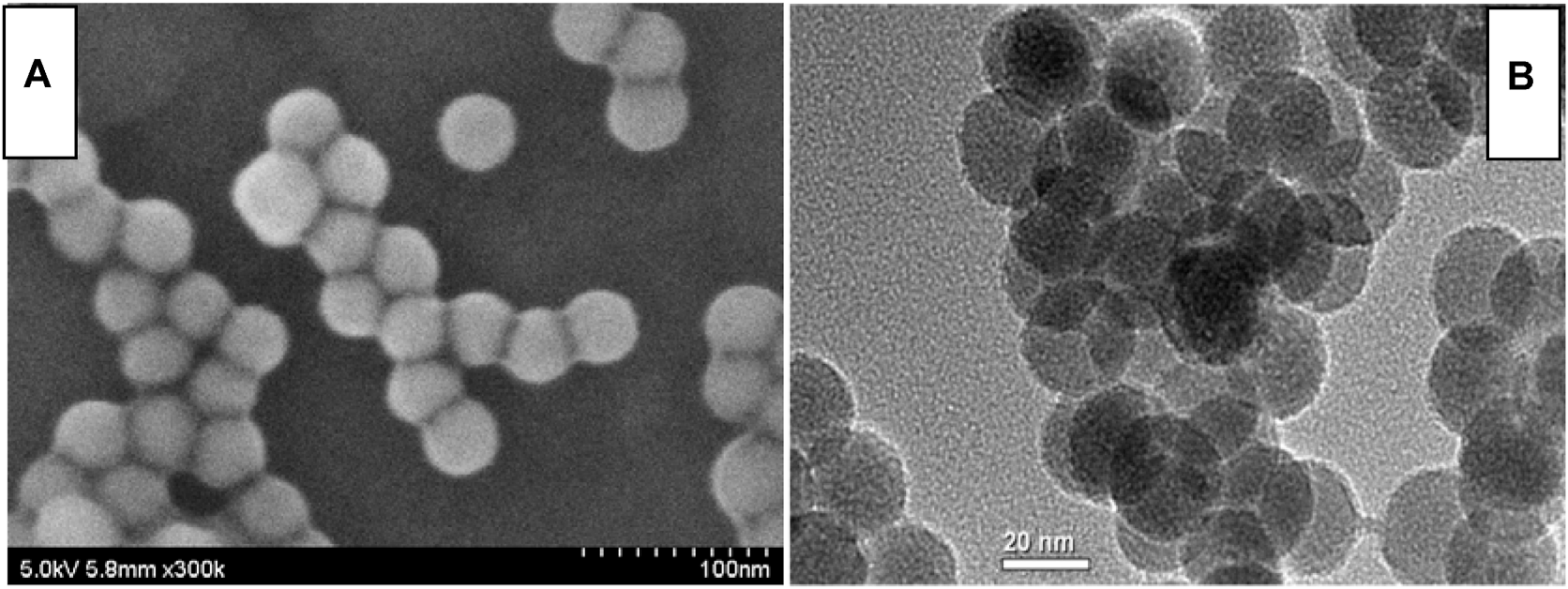
SEM **(A)** and TEM **(B)** of our up-conversion host modified by α-CD.

**FIGURE 2:**
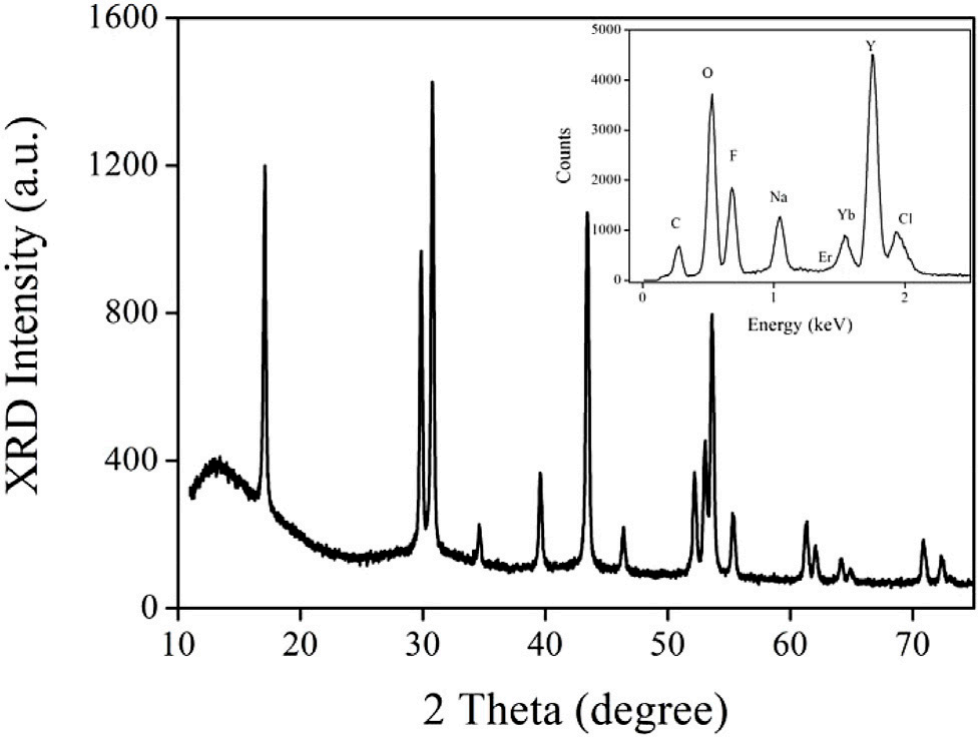
Wide angle XRD pattern of our up-conversion NaYF_4_ lattice. Inset: EDX spectrum of our up-conversion NaYF_4_ lattice.

The micromorphology of the as-synthesized up-converting NaYF_4_ lattice was evaluated using microscopy images. As demonstrated by Figure 1, all nanocrystals were spherical with an average diameter of ∼24 nm. Uniform distribution and smooth surface were detected, which confirms that sample morphology was hardly affected by the α-CD modification. The EDX spectrum of this up-conversion NaYF_4_ lattice suggests that it contained eight elements, as shown by the inset of Figure 2, including carbon, oxygen, fluorine, sodium, erbium, ytterbium, yttrium, and chlorine. The carbon and oxygen elements should be attributed to organic components in CD-NaYF_4_, such as cyclodextrin and OA. The Cl element was assigned to the rare earth chloride leftover. The remaining elements matched the elemental composition of the desired up-converting lattice. Figure 2 shows the wide angle XRD pattern of our up-conversion NaYF_4_ lattice. There were 15 sharp 2θ peaks which belonged to 2θ peaks of hexagonal NaYF_4_ (JCPDS 28-1192). Since there were no 2θ peaks coming from impurities or other phases, we concluded that ytterbium and erbium ions were trapped in the NaYF_4_ crystal cell. As a consequence, the excitation lattice NaYF_4_:Yb^3+^/Er^3+^ was constructed.

**FIGURE 3:**
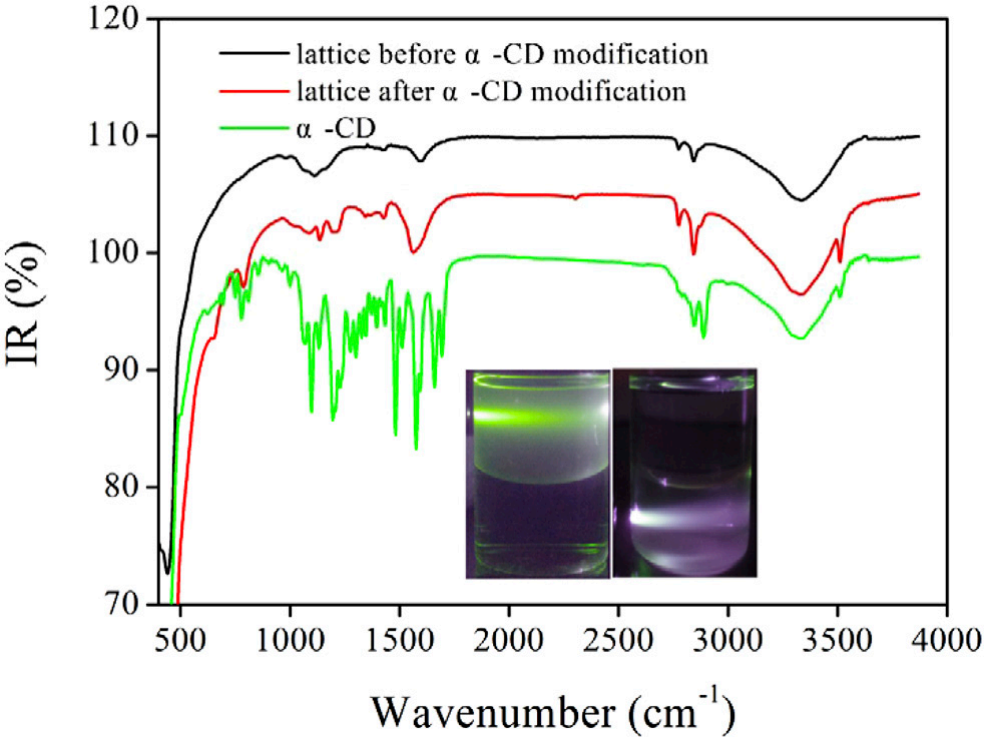
IR spectra of free α-CD and our excitation lattice before and after α-CD modification. Inset: photos of our excitation lattice in hexane/water-mixed solvent before and after α-CD modification (λ_ex_ = 980 nm).

IR spectra of the excitation lattice before/after cyclodextrin coating and free α-CD are compared in Figure 3. The IR spectrum of the excitation lattice before α-CD modification had vibration peaks centering at ∼2852 cm^−1^. They were attributed to coupled stretch vibrations of the C-OH group from oleic acid (Zhang et al., 2012). These bands were observed after α-CD modification, confirming that our excitation lattice was still covered by OA. Free α-CD had three characteristic IR peaks, which were 778 cm^−1^, 1097 cm^−1^, and 3,510 cm^−1^, respectively. The first one was due to coupled stretch vibrations of the C-C band. The second one was attributed to vibrations from C-O bands. While the last one belonged to the anti-symmetric vibration of the C-O-C band (Zhang et al., 2012). These three IR bands could all be traced in the IR spectrum of CD-NaYF_4_, suggesting successful cyclodextrin modification.

For a visual understanding on its hydrophily variation, the as-synthesized exciting lattice and CD-NaYF_4_ were individually dispersed in hexane/H_2_O solvent. As shown in Figure 3 (inset), before α-CD modification, nanoparticles were aggregated in a hexane layer (up) due to the hydrophobic surface. Green up-conversion light was observed when exposed to a 980 nm laser. After cyclodextrin coating, all nanoparticles were distributed in the bottom H_2_O phase, suggesting their hydrophilic surface. Up-conversion light was well observed, confirming that cyclodextrin coating had little effect on the emissive center.

### 3.3 Photophysical Feature of Our Up-Conversion Lattice and Sensor-1 and Sensor-2

#### 3.3.1 Energy Transfer *via* Spectral Overlap

**FIGURE 4:**
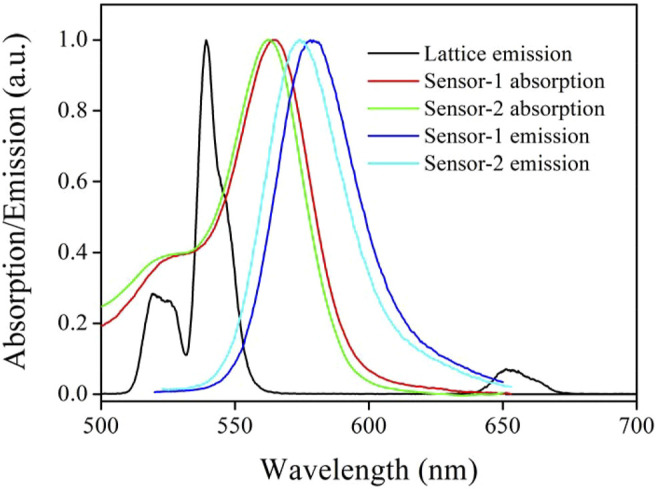
Emission and absorption spectra of our excitation lattice (in PBS, pH = 7.0, 5 mg in 10 ml, λ_ex_ = 980 nm) and two chemosensors (in CH_2_Cl_2_, 10 μM, with 1 eq. of Cys).

To check the energy transfer (ET) from CD-NaYF_4_ to Sensor-1 and Sensor-2, chemosensor absorption and CD-NaYF_4_ emission spectra are compared in Figure 4. Upon 980 nm radiation, our excitation lattice showed multiple emission bands, which were 519, 539, and 651 nm, respectively. Their wavelengths matched ^2^H_11∕2_→^4^I_15∕2_, ^4^S_3∕2_→^4^I_15∕2_, ^4^F_9∕2_→^4^I_15∕2_ emissions of Er^3+^ ions, confirming the successful preparation of our up-conversion lattice (Li and Zhang, 2006; Zhang et al., 2012). The two chemosensor absorption spectra were quite similar, owing to their similar molecular composition. Sharp absorption bands were observed at 565 nm for Sensor-1 and 563 nm for Sensor-2. The chemosensor absorption band covered CD-NaYF_4_ major emission bands well, as shown in Figure 4, which indicates the possibility of ET between CD-NaYF_4_ and Sensor-1 and Sensor-2. Given Cys (1 eqv.), our chemosensors take their emissive structure, with emission wavelengths of 580 and 575 nm, respectively. Sulfur modification caused a slight red shift (Wang et al., 2011; Zhang et al., 2012).

#### 3.3.2 ET Radius

The ET radius (R_0_) between CD-NaYF_4_ and Sensor-1 and Sensor-2 was defined by the below formulas. Q_0_, J, κ^2^, n_d_, N_A_, λ, f_d_(λ), and ε_A_(λ) in Eq. 1 and Eq. 2 denote NaYF_4_ yield (3.0%, following Boyer’s report), spectral overlap integral, mutual molecular orientation, solvent refraction index, Avogadro number, wavelength, host emission, and chemosensor absorbance efficiency, respectively (Xie et al., 2009; Boyer and van Veggel, 2010; Zou et al., 2014). R_0_ values were calculated as 22 Å by Eq. 1 and Eq. 2. This value (22 Å) was higher than traditional values. Its causation should be the high spectral overlap between CD-NaYF_4_ emission and Sensor-1 and Sensor-2 absorption (Wang et al., 2011; Cui and Zhang, 2014; Zou et al., 2014). As a consequence, CD-NaYF_4_ should be able to transfer its energy efficiently to Sensor-1 and Sensor-2 even in a highly dispersed solution.

$$R_{0}^{6}=\frac{9Q_{0}k^{2}J\left(\ln10\right)}{128\pi^{5}n_{d}^{4}N_{A}}$$ (1)

$$J=\int f_{D}\left(\lambda \right)\varepsilon_{A}\left(\lambda \right)\lambda^{4}d\lambda$$ (2)

#### 3.3.3 Emission Decay Dynamics Analysis

**FIGURE 5:**
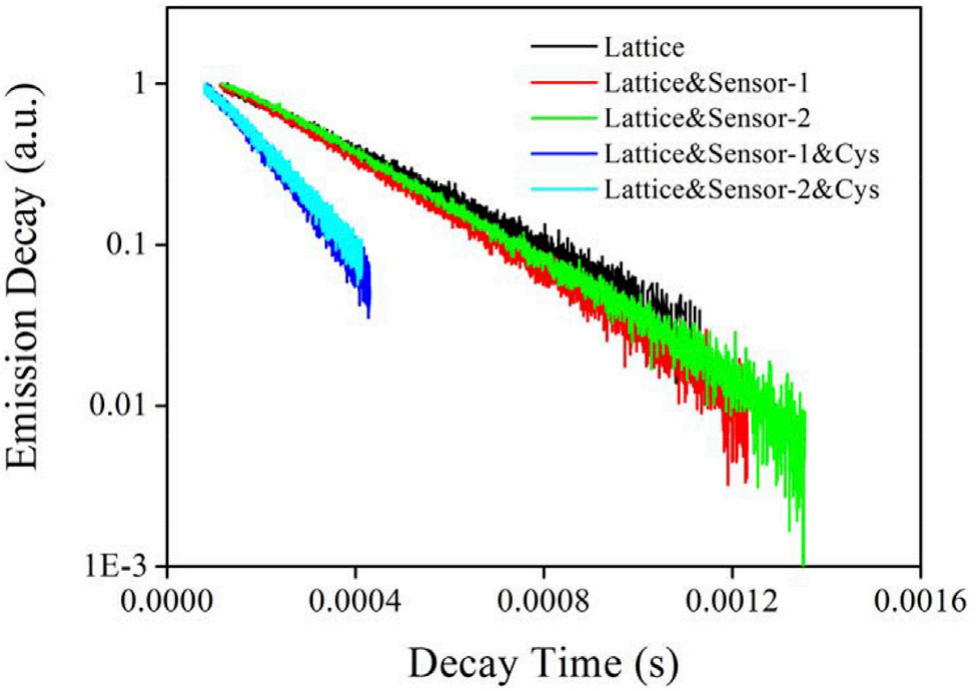
Emission decay lifetimes of our excitation lattice (539 nm) upon the presence chemosensors and Cys (in PBS, pH = 7.0, 5 mg in 10 ml for lattice, 10 μM for chemosensors, 10 μM for Cys, λ_ex_ = 980 nm).

Emission dynamics of CD-NaYF_4_ (539 nm) were monitored when exposed to Sensor-1 and Sensor-2 and cysteine so that the energy transfer between them could be further understood. In Figure 5, the intrinsic lattice presented a long-lived state of 311 μs which was slightly longer than literature values (Zhang et al., 2012). Its linear decay dynamics indicated that Er(III) ions had been highly dispersed in CD-NaYF_4_ with no difference. Sensor-1 and Sensor-2 quench lattice emission was very slim, with a decay state of 271 μs for Lattice:Sensor-1 and 286 μs for Lattice:Sensor-2. Corresponding ET efficiency (η) is defined by Eq. 3, and found to be 12.9 and 8.0%, respectively. Here τ is the lattice decay lifetime and ' denotes no energy acceptor. These η values were low, which means the ET from CD-NaYF_4_ to pure Sensor-1 and Sensor-2 was inefficient. In other words, Sensor-1 and Sensor-2 incorporated spirolactam geometry and were not open to CD-NaYF_4_ energy transfer. The presence of cysteine (1 eqv.) made Sensor-1 and Sensor-2 take an emissive geometry and open for lattice emission. Consequently, lattice emissive dynamics were affected, showing lifetimes of 146 and 161 μs, respectively. Their η values were increased to 53.1 and 48.2%, respectively. The improved ET from CD-NaYF_4_ to Sensor-1 and Sensor-2 was thus confirmed. The sulfur substituent slightly increased chemosensor absorption intensity, so the η value of Lattice:Sensor-1 was higher than that of Lattice:Sensor-2.

$$\eta =1-\tau^{\prime }/\tau$$ (3)

#### 3.3.4 Job’s Plot and Binding Stoichiometry

**FIGURE 6:**
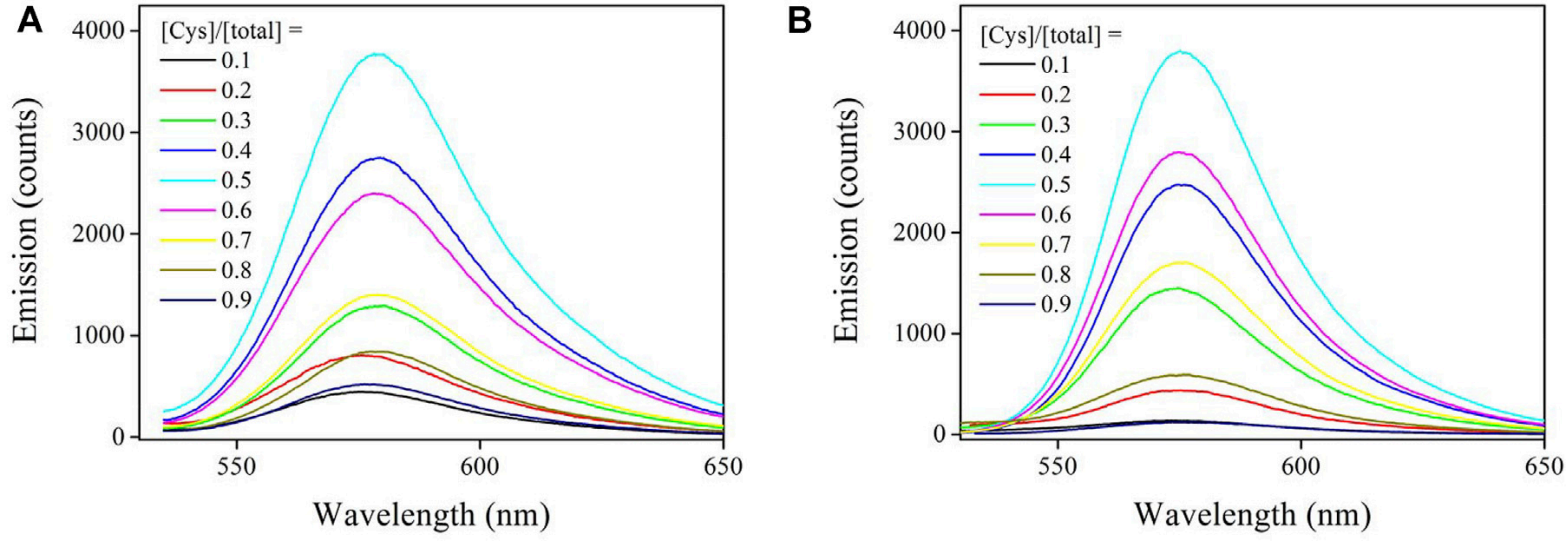
**(A)** Emission spectra of Sensor-1 and Cys. Their total concentration is fixed as 10 μM, then the Cys molar fraction is increased from 0.1 to 0.9. **(B)** Emission spectra of Sensor-2 and Cys. Their total concentration is fixed as 10 μM, then the Cys molar fraction is increased from 0.1 to 0.9.

The Job plot experiment was applied to find the binding stoichiometry between Sensor-1, Sensor-2, and cysteine. Here, their total concentration was fixed (10 μM). By gradually increasing the cysteine molar ratio, their emission spectra are compared in Figure 6. Clearly, chemosensor emission was greatly increased by the presence of Cys. Upon a Cys molar fraction of 0.5, chemosensor emission intensity was maximized. Both increasing or decreasing the cysteine molar ratio tended to compromise chemosensor emission intensity. This result suggests that Sensor-1 and Sensor-2 coordinated with cysteine under binding stoichiometry of 1 *vs.* 1. A schematic presentation is shown by Eq. 4, where K_s_ denotes the association constant. This uncomplicated binding mechanism may give a linear sensing response towards Cys concentration variation, which will be later proved.

$$\text{Sensor}-1/2+Cys\overset{Ks}{↔}\text{Sensor}-1/2:Cys$$ (4)

**FIGURE 7:**
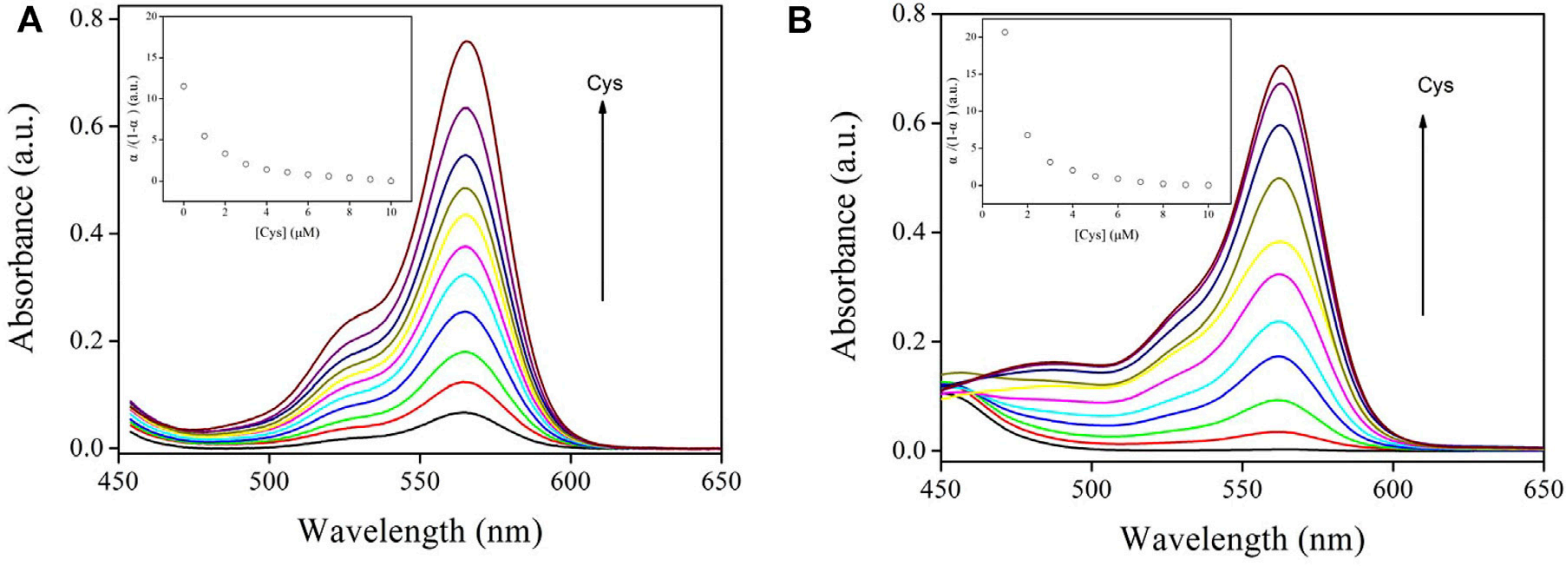
**(A)** Sensor-1 absorption spectra against various Cys concentrations (in PBS, 10 μM, pH = 7.0). Inset: α/(1-α) *vs.* [Cys] characteristics. **(B)** Sensor-2 absorption spectra against various Cys concentrations (in PBS, 10 μM, pH = 7.0). Inset: α/(1-α) *vs.* [Cys] characteristics.

It should be pointed out that Job plots can only be used as an “after the fact” verification once the Ks has been established based on titration experiment data, according to Hibbert’s report (Brynn Hibbert and Thordarson, 2016). To confirm the validity of the above Job plots, the K_s_ value was fitted based on an absorption titration experiment, as depicted in Eqs. 5, 6. Here A_T_ is the absorbance without Cys and A_0_ is absorbance with 100% Cys (Zhang et al., 2007; Zhang et al., 2012). It is observed in Figure 7 that Sensor-1 and Sensor-2 absorbance increased with increasing cysteine concentration, which means a complexation procedure between chemosensors and Cys. Corresponding K_s_ values were obtained as 1.80 × 10^5^ M^−1^ and 0.59 × 10^5^ M^−1^, respectively. These values were higher than traditional ones, suggesting that Sensor-1 and Sensor-2 have improved their binding performance with Cys (Li and Zhang, 2006; Liu et al., 2008; Zhou et al., 2012; Zou et al., 2014). In addition, it was found that the S substituent greatly increased the K_s_ value of Sensor 1, compared to that of Sensor 2. This observation is explained by the higher binding energy between sulfur and cysteine (Wang et al., 2011; Cui and Zhang, 2014; Zou et al., 2014).

$$\frac{\alpha }{1-\alpha }=\frac{1}{Ks\left[\left(Cys\right)\right]}$$ (5)

$$\alpha =\frac{A_{T}-A}{A_{T}-A_{0}}$$ (6)

The response time of these chemosensors towards Cys was explored by monitoring their emission intensity after adding Cys. It is observed from Supplementary Figure S1 that both chemosensors increased their emission intensity in the first 3 min quickly, then their emission intensity was gradually increased, and finally remained constant after 4 min. A quick sensing response of these chemosensor towards Cys was thus confirmed.

### 3.4 Sensing Performance of CD-NaYF_4_ and Sensor-1 and Sensor-2 Systems

#### 3.4.1 Emission Spectra

**FIGURE 8:**
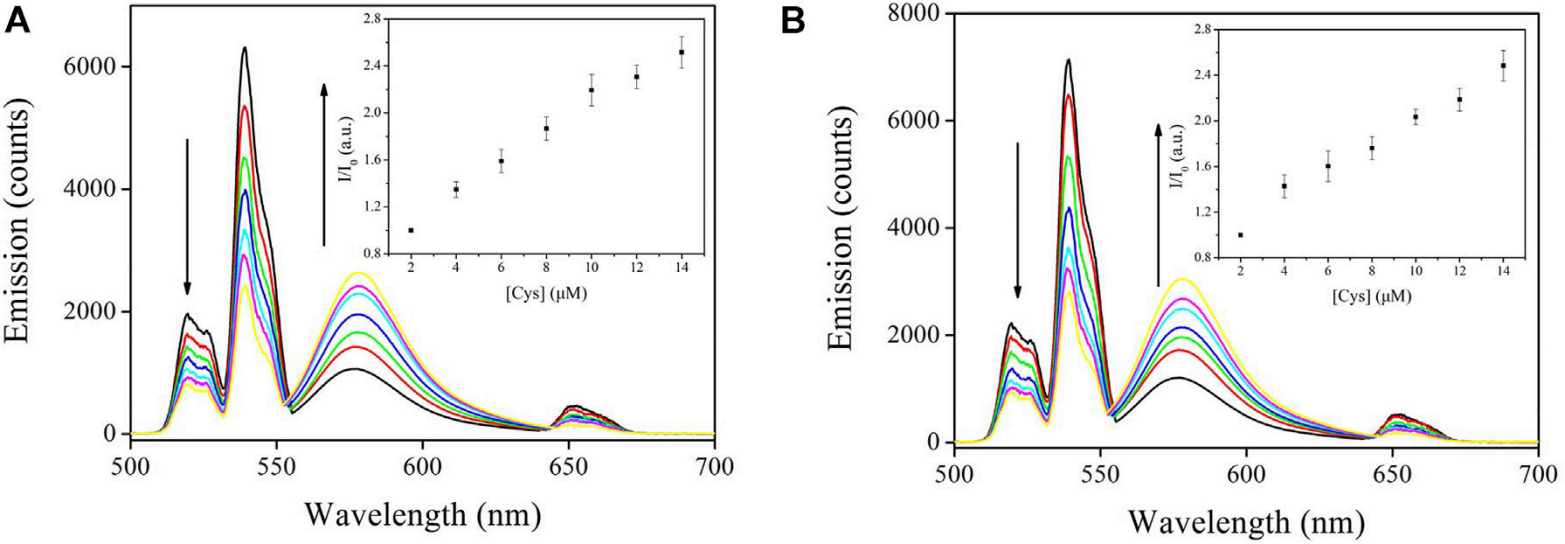
**(A)** Emission spectra of Lattice:Sensor-1 upon various Cys concentrations using NaYF_4_:Yb^3+^/Er^3+^ nanocrystals as the excitation host (in PBS, pH = 7.0, 5 mg in 10 ml for host, 10 μM for Chemosensor 1, λ_ex_ = 980 nm). Inset: corresponding I/I_0_ *vs.* [Cys] variation. **(B)** Emission spectra of Lattice:Sensor-2 upon various Cys concentrations using NaYF_4_:Yb^3+^/Er^3+^ nanocrystals as the excitation host (in PBS, pH = 7.0, 5 mg in 10 ml for host, 10 μM for Chemosensor 2, λ_ex_ = 980 nm). Inset: corresponding I/I_0_ *vs.* [Cys] variation.

The emission spectral response of CD-NaYF_4_:Sensor-1 and Sensor-2 when exposed to increasing cysteine concentration is given in Figure 8. Unsurprisingly, all CD-NaYF_4_ emission bands were weakened by cysteine. In the meanwhile, Sensor-1 and Sensor-2 emission was enhanced a lot. At a Cys concentration of 14 μM, the emission intensity of CD-NaYF_4_:Sensor-1 was 2.51-fold higher. While that of CD-NaYF_4_:Sensor-2 was 2.48-fold higher than its initial value. Emission wavelength and band shape of CD-NaYF_4_:Sensor-1 and Sensor-2 systems were similar to those of free chemosensors, suggesting that Sensor-1 and Sensor-2 were well preserved after meeting the CD-NaYF_4_ excitation lattice.

As for the final up-converting emission band (∼650 nm), it is usually reported to be unaffected by the energy quencher and applied as an inner standard for fluorescence titration (Li and Zhang, 2006; Liu et al., 2008; Wang et al., 2011; Zhou et al., 2012; Cui and Zhang, 2014; Zou et al., 2014). But this was not the case in this work. For both CD-NaYF_4_:Sensor-1 and Sensor-2 systems, all CD-NaYF_4_ emission bands were decreased by cysteine, including the up-converting band at 651 nm. This result means that the ^4^F_9∕2_ state of Er(III) transferred its energy to our chemosensors like ^2^H_11∕2_ and ^4^S_3∕2_. Taking our above analysis on emission decay dynamics, it was concluded that the dominant ET procedure from CD-NaYF_4_ to Sensor-1 and Sensor-2 was a Forester procedure.

#### 3.4.2 Stern–Volmer Plots

In virtue of their simple binding mechanism (1 *vs.* 1) between Sensor-1 and Sensor-2 and cysteine, the chemosensor emission response against cysteine concentration should be discussed using a Stern–Volmer plot, as described by Eq. 7. Here I is the Sensor-1 and Sensor-2 intensity form, 0 means no energy acceptor, [Cys] stands for cysteine concentration, and K_sv_ is the SV constant. Linear working curves were fitted, as depicted in Figure 8 (inset). Our chemosensors were found to be superior to sensors from literature since they followed a linear sensing performance (Li and Zhang, 2006; Liu et al., 2008; Xie et al., 2009; Boyer and van Veggel, 2010; Zhou et al., 2012; Brynn Hibbert and Thordarson, 2016; Guan et al., 2016). We attributed the causation to the uncomplicated binding mechanism mentioned in *Job’s Plot and Binding Stoichiometry*. In addition, α-CD modification on our excitation lattice ensured our NaYF_4_ nanocrystals were uniformly dispersed, resulting in linear sensing behavior of our chemosensors. Linearity of Lattice:Sensor-1 was slightly better than that of Lattice:Sensor-2. It appears that the chemosensor sulfur modification improved the linearity of the working curve as well. Corresponding limit of detection (LOD) values were determined as 2.7 μM for Lattice:Sensor-1 and 2.8 μM for Lattice:Sensor-2. These LOD values were much lower than the normal Cys concentration in human serum (15.2 ± 0.2 mM, real world Cys concentration) (Guan et al., 2016). Considering the effective working region of these chemosensors (2–14 μM), human serum samples should be diluted 1000 times to meet the optimal sensitivity of these chemosensors (∼2.5, target sensitivity).

$$I/I_{0}=1+K_{sv}\left[Cys\right]$$ (7)

K_sv_ values were fitted to 1.26 × 10^5^ M^−1^ and 1.14 × 10^5^ M^−1^, respectively. These values improved compared to traditional values (Li and Zhang, 2006; Liu et al., 2008; Xie et al., 2009; Boyer and van Veggel, 2010; Zhou et al., 2012; Brynn Hibbert and Thordarson, 2016; Guan et al., 2016). We thus came to the conclusion that terephthalaldehyde modification and sulfur modification led to better sensing performance. In this paper, sensitivity was calculated by the ratio of I/I_0_ at a cysteine concentration of 14 μM. Sensitivity values were consequently determined as 2.51 and 2.48, respectively. There was no obvious difference between sensitivity values of our chemosensors. It seems that chemosensor sulfur modification just affected the linearity of the working curve but exerted little effect on sensitivity. Nevertheless, our sensitivity values were far from satisfactory (Li and Zhang, 2006; Wang et al., 2011; Zhang et al., 2012; Guan et al., 2016). For further improvement, intrinsic emission intensity (I_0_) should be minimized. According to Eq. 7, the intrinsic emission intensity (I_0_) was the chemosensor emission intensity in the absence of Cys. The observation of intrinsic chemosensor emission intensity suggests that some chemosensor molecules started their structural transformation from a spirolactam structure (non-emissive) to a delocalized xanthene structure (emissive) without the help of Cys. Considering that both chemosensors suffered from such high intrinsic emission intensity, the high I_0_ may be connected to excitation source. The oleic acid on the NaYF_4_:Yb^3+^/Er^3+^ surface may be responsible for the chemosensor structural transformation since an acidic environment leads to rhodamine structural transformation as well (Wang et al., 2011; Zhang et al., 2012). To mitigate intrinsic chemosensor emission intensity, these oleic acid chains on NaYF_4_:Yb^3+^/Er^3+^ surface should be completely removed.

#### 3.4.3 Photostability Comparison

**FIGURE 9:**
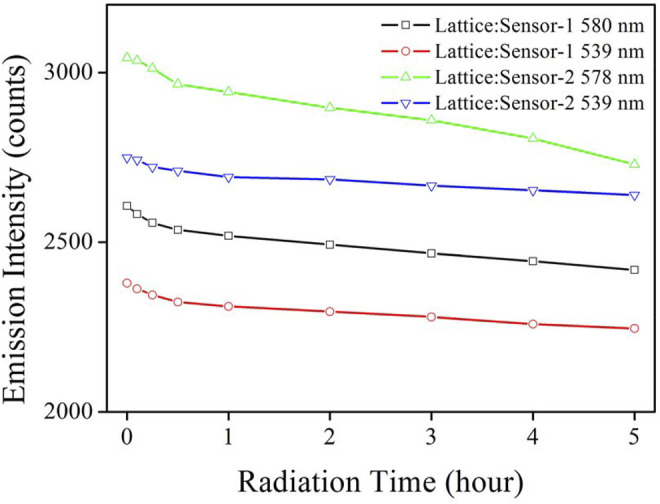
Emission intensity monitoring of Lattice:Chemosensor systems under continuous radiation (in PBS, pH = 7.0, 5 mg in 10 ml for host, 10 μM for chemosensors, 10 μM for Cys, λ_ex_ = 980 nm).

To confirm the improved chemosensor photostability in this work, emission intensity monitoring was performed on our Lattice:Chemosensor systems under continuous radiation and shown in Figure 9. Since luminescence intensity is a very relevant parameter for photostability, the initial luminescence intensity of both sensing systems in Figure 9 was adjusted to be the same as the emission intensity used for the measurement of Figure 8. As for CD-NaYF_4_, its emission intensity (539 nm) remained stable during 5 h of radiation exposure. We attributed its stability to its strong NaYF_4_ structure (Li and Zhang, 2006; Liu et al., 2008; Zhou et al., 2012). Its minor decrease should be explained by the particle aggregation. Our chemosensors, however, showed much more obvious emission photobleaching, especially for Sensor-2. It seems that the laser heating effect still struck the organic components. On the other hand, their photobleaching effect was much weakened, compared to that of UV-excited chemosensors (Johansson et al., 1993; Zhang et al., 2010; Saha et al., 2012a; Saha et al., 2012b; Guan et al., 2016). Over 92.8% of its initial emission value was preserved by Lattice:Sensor-1 after 5 h of continuous radiation. As for Lattice:Sensor-2, 89.7% of its initial value was recorded after 5 h of continuous radiation. The sulfur substituent effect was thus found to be positive to improve chemosensor photostability. Consequently, it was concluded that the utilization of the up-conversion lattice greatly improved the photostability of the Lattice:Chemosensor systems. To explore the service life of these chemosensors, their sensitivity was monitored upon continuous radiation time. It is shown in Supplementary Figure S2 that Lattice:Sensor-1 preserved 95% of its initial sensitivity value for 3 h of continuous radiation, while Lattice:Sensor-2 preserved 95% of its initial sensitivity value for 2 h of continuous radiation. As a consequence, their service life values were 3 and 2 h, respectively.

#### 3.4.4 Selectivity Analysis

**FIGURE 10:**
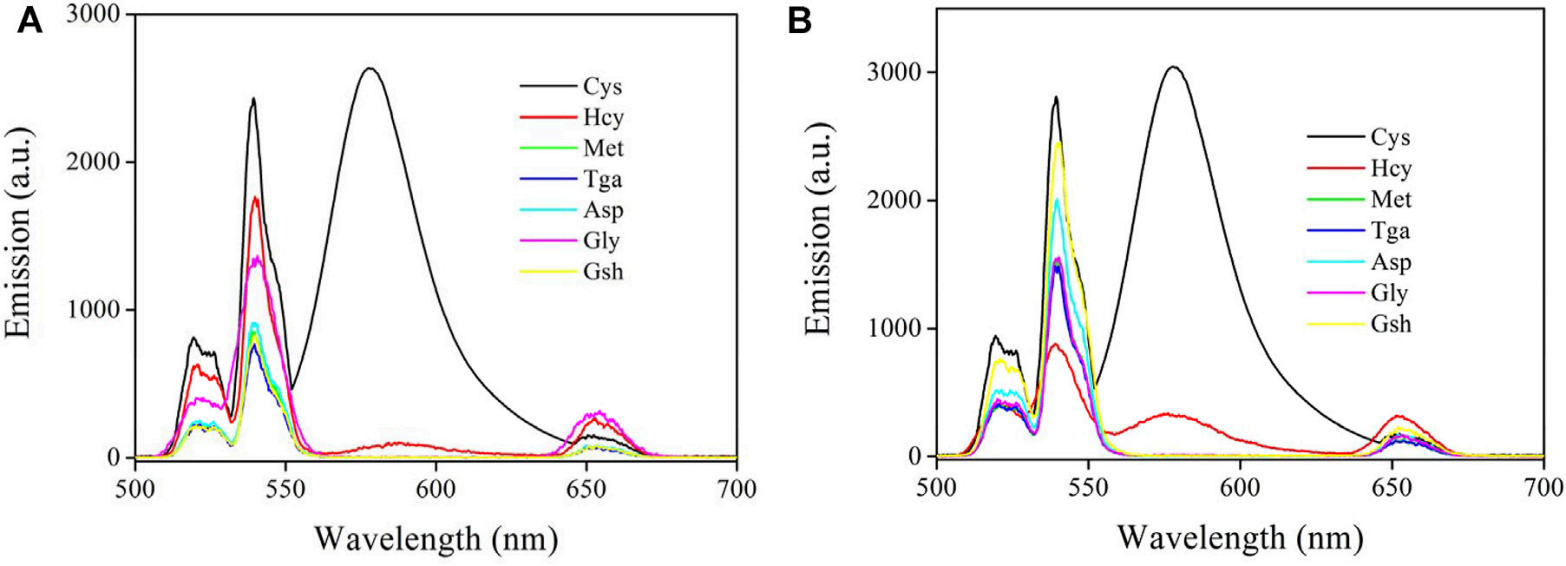
**(A)** Emission spectra of Sensor-1 under Cys and several competing amino acids and thiols (20 μM) (in PBS, pH = 7.0, 5 mg in 10 ml for host, 10 μM for Sensor-1, λ_ex_ = 980 nm). Homocysteine (Hcy), methionine (Met), thioglycolic acid (Tga), aspartic acid (Asp), glycine (Gly), and glutathione (Gsh). **(B)** Emission spectra of Sensor-2 under Cys and several competing amino acids and thiols (20 μM) (in PBS, pH = 7.0, 5 mg in 10 ml for host, 10 μM for Sensor-2, λ_ex_ = 980 nm). Homocysteine (Hcy), methionine (Met), thioglycolic acid (Tga), aspartic acid (Asp), glycine (Gly), and glutathione (Gsh).

The selectivity of CD-NaYF_4_:Sensor-1 and Sensor-2 for cysteine was due to the specific signaling of Sensor-1 and Sensor-2 for cysteine in a complicated environment full of competing species. Their emission spectra upon cysteine and several competing species are given in Figure 10. Unsurprisingly, cysteine led to enhanced chemosensor emission. Nevertheless, our chemosensors showed no obvious response towards nearly all competing amino acids and thiols, with an exception of homocysteine. Owing to their nearly identical molecules, homocysteine (Hcy) and cysteine can both enhance chemosensor emission. Homocysteine shows a less effective effect, however. It has been reported by literature that a rhodamine-derived chemosensor usually finishes its recognition procedure by constructing a five- (cysteine) or six-membered ring (homocysteine) [(Johansson et al., 1993; Zhao et al., 2010; Saha et al., 2012b)]. Generally speaking, a five-membered ring is not as robust as a six-membered one. But its cyclization dynamics are faster than that of a six-membered ring. As a consequence, Sensor-1 and Sensor-2 showed good selectivity for cysteine over Hcy through a dynamic mechanism. In addition, Sensor-1 selectivity was found to be improved compared to Sensor-2, suggesting that the sulfur substituent effect was positive to improve chemosensor selectivity. This is because the sulfur atom has a low affinity for competing species due to its higher tension when constructing a six-membered ring.

The remaining competing species showed no obvious interference on Sensor-1 and Sensor-2, but quenched lattice emission obviously. These competing acids and thiols just absorb and quench lattice emission with no emission turn-on effect. This means that Sensor-1 and Sensor-2 are still taking their non-fluorescent structure. Owing to their unsuitable geometric structures, these competing acids and thiols all fail to trigger the emission turn-on structural transformation. In this case, good selectivity was realized by our chemosensors through a dynamic mechanism, which favors practical applications. On the other hand, it should be pointed out that some other environmental factors may affect these chemosensors and their emission intensity. For example, excess protons (acidic condition) may trigger the emission turn-on structural transformation, causing increased emission intensity. Some transition metal ions, such as Cu(II) and Hg(II), may trigger such emission turn-on structural transformation as well (Wang et al., 2011; Zhang et al., 2012). To get a precise result, these negative factors should be considered and eliminated.

## 4 Conclusion

Briefly, this paper reported two optical sensing platforms for cysteine detection. The up-converting nanoparticles were modified by cyclodextrin and applied as an excitation host. Rhodamine-like molecules were synthesized as probes. Full characterization on these nanocrystals and chemosensors was performed to confirm their identity. An energy transfer procedure from these nanocrystals and rhodamine sensors was established via their spectroscopic analysis and emissive decay dynamics comparison. The binding dynamics of our chemosensors for Cys were revealed to have uncomplicated recognition with a stoichiometric ratio of 1 *vs.* 1. The resulting sensing systems exhibited enhanced emission for cysteine with linear response and selectivity. Sulfur modification on our chemosensors was shown to be effective in improving their selectivity and photostability. Nevertheless, chemosensor emission residue should be decreased aiming at better sensitivity.

There is still a disadvantage of these Lattice:Chemosensor systems because of the following two reasons. First, the effective working region of these chemosenosrs (2–14 μM) is much lower than the normal Cys concentration in human serum (15.2 ± 0.2 mM). In this case, human serum samples should be diluted 1000 times to meet the optimal sensitivity of these chemosensors (∼2.5). Second, the working curves of these chemosensors are just linear-like ones, with uncertainties. Theoretically, sample Cys concentration can be determined by these working curves. But, before so doing, serum samples must be diluted, causing uncertainty. As a consequence, we cautiously say that these Lattice:Chemosensor systems in this work are able to detect a fixed amount of Cys in human body, but are not good at doing it.

For further research effort, their potential application in biological application should be verified, considering the up-converting excitation nanocrystals and good selectivity of these chemosensors. There are problems to be solved, though. First, as mentioned above, the effective working region of these chemosensors is 2–14 μM, while the normal Cys concentration in human serum is 1000 times higher (15.2 ± 0.2 mM). Thus, the doping ratio of the excitation source and chemosensor should be adjusted to meet the normal Cys concentration in human serum. Second, although the up-converting nanocrystals have been coated and modified by α-CD, there are still oleic acid chains on their surface. These oleic acid chains result in an acidic environment around excitation nanocrystals, leading to high intrinsic chemosensor emission intensity. In addition, this acidic environment may harm bio-samples, compromising bio-imaging. As a consequence, before practical bio-imaging, these oleic acid chains should be completely removed.

## Data Availability

The datasets presented in this study can be found in online repositories. The names of the repository/repositories and accession number(s) can be found in the article/Supplementary Material.
