# Supplementary material for: Up-Converting Nanocrystals Modified With Fluorescent Markers for the Detection of Amino Acids: Preparation, Characterization, and Sensing Performance
Source: Front Chem. 2022 Mar 21;10:859963. doi: 10.3389/fchem.2022.859963 (PMC8978546; doi:10.3389/fchem.2022.859963)

Figure S1. Emission intensity monitoring of Sensor-1 and Sensor-2 and Cys (1:1) in PBS, pH = 7.0.


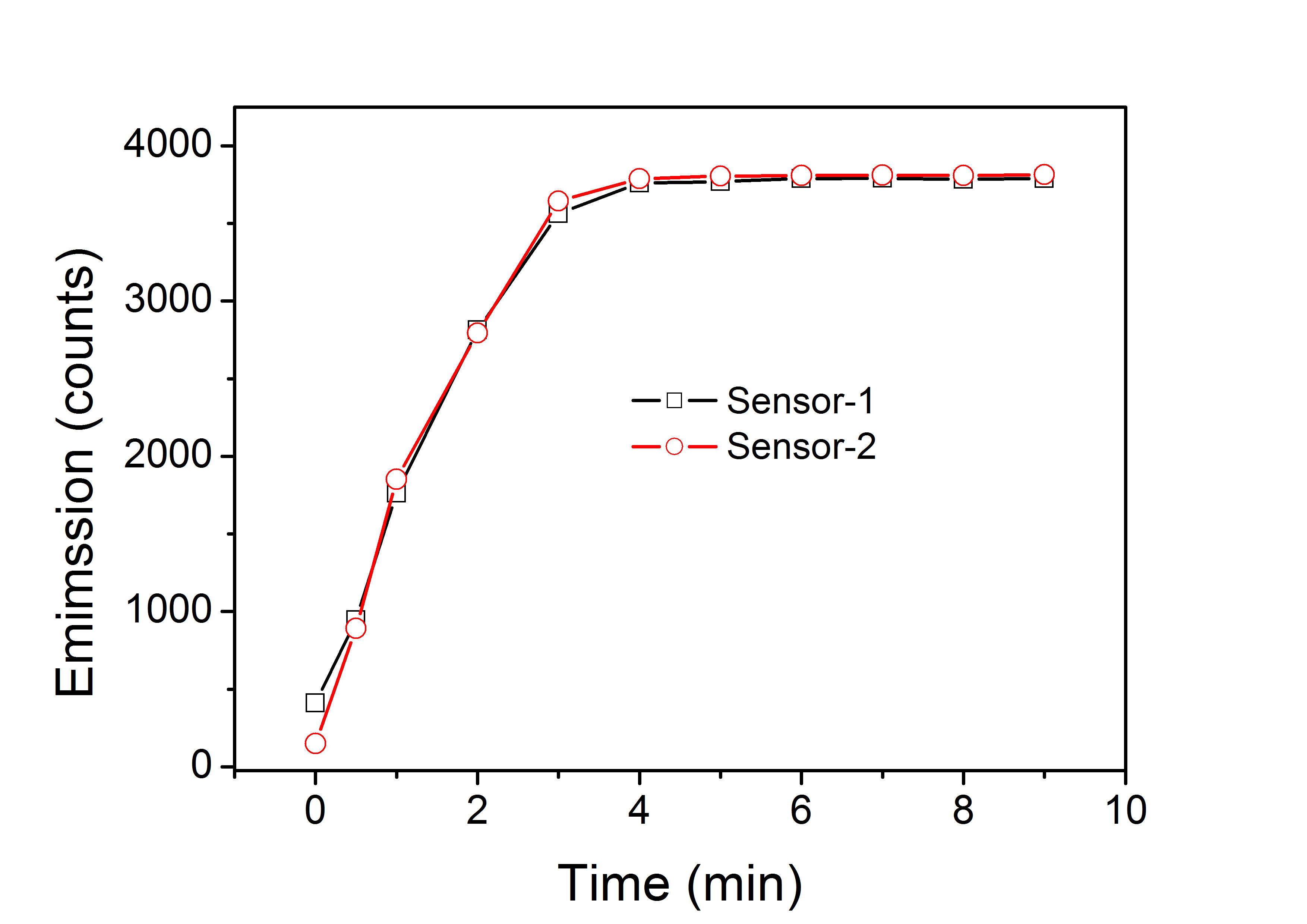


Figure S2. Sensitivity monitoring of Lattice:Sensor systems upon continuous radiation.


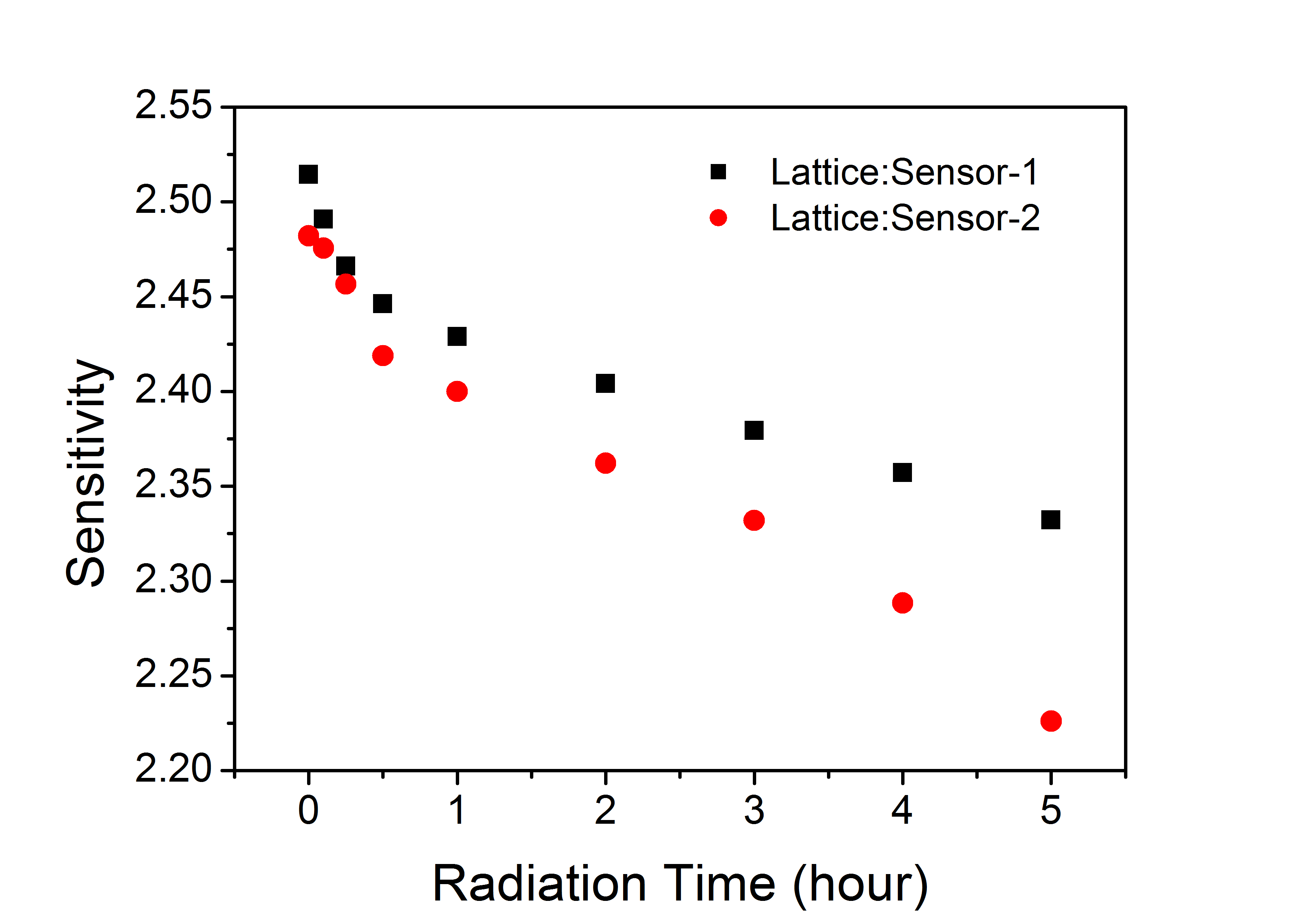

Supplement: Supplementary file 1 [file DataSheet1.docx]
